# (1-Phenyl­sulfonyl-1*H*-indol-2-yl)(thio­phen-2-yl)methanone

**DOI:** 10.1107/S1600536811005666

**Published:** 2011-02-26

**Authors:** C. KamalaKumar, V. Dhayalan, A. K. Mohanakrishnan, V. Balasubramanian, V. Manivannan

**Affiliations:** a Department of Chemistry, AMET University, Kanathur, Chennai 603 112, India; b Department of Organic Chemistry, University of Madras, Guindy Campus, Chennai 600 025, India; c Department of Research and Development, PRIST University, Vallam, Thanjavur 613 403, Tamil Nadu, India

## Abstract

The crystal studied of the title compound, C_19_H_13_NO_3_S_2_, was found to be a non-merohedral twin with a domain ratio of 0.877 (3):0.123 (3). There are two independent mol­ecules in the asymmetric unit. The dihedral angles between the mean plane of the indole ring system and the phenyl­sulfonyl ring are 71.67 (13) and 71.95 (13)° in the two mol­ecules while  the indole unit and the thiophene ring make dihedral angles of 54.91 (12) and 56.92 (13)° in the two molecules. The crystal packing is stabilized by weak C—H⋯π inter­actions.

## Related literature

For biological activity of chromenopyrrole, see: Ma *et al.* (2001 ▶); Zhao *et al.* (2002 ▶); Zhou *et al.* (2006 ▶); Rajeswaran *et al.* (1999 ▶); For related structures, see: Chakkaravarthi *et al.* (2007 ▶); Gunasekaran *et al.* (2009 ▶); Saravanan *et al.* (2010 ▶).
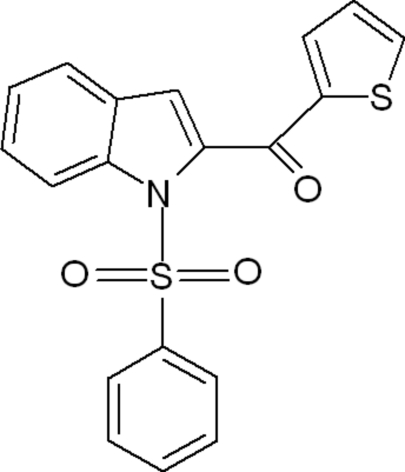


## Experimental

### 

#### Crystal data

C_19_H_13_NO_3_S_2_
*M* *_r_* = 367.42
Triclinic, 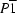

*a* = 9.3605 (5) Å
*b* = 10.8455 (5) Å
*c* = 17.5482 (9) Å
α = 88.716 (3)°
β = 80.425 (2)°
γ = 71.467 (2)°
*V* = 1664.68 (15) Å^3^
*Z* = 4
Mo *K*α radiation
μ = 0.34 mm^−1^
*T* = 295 K
0.35 × 0.25 × 0.20 mm

#### Data collection

Bruker Kappa APEXII CCD diffractometer
Absorption correction: multi-scan (*SADABS*; Sheldrick, 1996 ▶) *T*_min_ = 0.924, *T*_max_ = 0.951
36289 measured reflections
8039 independent reflections
6195 reflections with *I* > 2σ(*I*)
*R*_int_ = 0.040

#### Refinement

*R*[*F* ^2^ > 2σ(*F* ^2^)] = 0.077
*wR*(*F* ^2^) = 0.245
*S* = 1.07
8039 reflections
453 parameters
H-atom parameters constrained
Δρ_max_ = 0.55 e Å^−3^
Δρ_min_ = −0.37 e Å^−3^

### 

Data collection: *APEX2* (Bruker, 2004 ▶); cell refinement: *SAINT* (Bruker, 2004 ▶); data reduction: *SAINT*; program(s) used to solve structure: *SHELXS97* (Sheldrick, 2008 ▶); program(s) used to refine structure: *SHELXL97* (Sheldrick, 2008 ▶); molecular graphics: *PLATON* (Spek, 2009 ▶); software used to prepare material for publication: *SHELXL97*.

## Supplementary Material

Crystal structure: contains datablocks global, I. DOI: 10.1107/S1600536811005666/bt5452sup1.cif

Structure factors: contains datablocks I. DOI: 10.1107/S1600536811005666/bt5452Isup2.hkl

Additional supplementary materials:  crystallographic information; 3D view; checkCIF report

## Figures and Tables

**Table 1:** Hydrogen-bond geometry (Å, °) *Cg* is the centroid of the C20–C25 ring.

*D*—H⋯*A*	*D*—H	H⋯*A*	*D*⋯*A*	*D*—H⋯*A*
C17—H17⋯*Cg*8^i^	0.93	2.88	3.693 (6)	147

Symmetry code: (i) 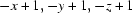.

**Fig. 1.**
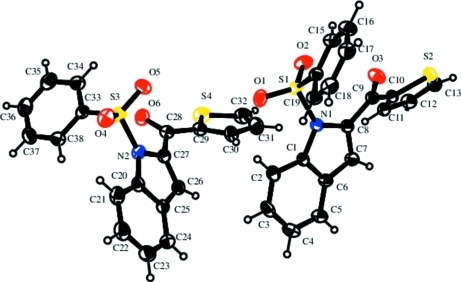
The molecular structure of the title compound, with atom labels and 30% probability displacement ellipsoids for non-H atoms.

## supplementary crystallographic information

### Comment

Indole derivatives are found to possess anticancer, antimalarial and antihypertensive activities (Ma *et al.*, 2001; Zhou *et al.*, 2006; Zhao *et al.*, 2002). In addition, Indoles have been proved to display high aldose reductase inhibitory activity (Rajeswaran *et al.*, 1999).

The geometric parameters of the title molecule (Fig. 1) agree well with reported similar structure(Chakkaravarthi *et al.*, 2007; Gunasekaran *et al.*, 2009; Saravanan *et al.*, 2010). The compound is non-merohedrally twinned, the suggested transformation matrix is (-1 0 0, 0 - 1 0, -0.664 0.110 1). The dihedral angle between the nine membered indole moiety and the thiophene ring is 54.91 (12) ° for molecule (I) and 56.92 (13) ° for molecule (II) respectively. The torsion angles O1—S1—N1—C1 and O2—S1—N1—C8 in molecule (I), O4—S3—N2—C20 and O5—S3—N2—C27 in molecule (II) [-9.8 (4) ° and 27.7 (4) ° for molecule (I), 9.1 (4) ° and -27.2 (4) ° for molecule (II), respectively] indicates the *syn* conformation of the sulfonyl moiety.

The sum of bond angles around N1 and N2 are 358.9 (3) ° and 358.6 (3) ° respectively, indicates the *sp^2^* hybridization state of atoms N1 and N2. The molecular structure is stabilized by weak intramolecular C—H···O interactions and the crystal packing is stabilized by weak C—H···π [C17—H17···*Cg*8(1 - *x*, 1 - *y*, 1 - *z*) distance of 3.693 (6)Å (*Cg*8 is the centroid of the ring defined by the atoms C20—C25)] interactions.

### Experimental

To a solution of *N*-(2-Formylphenyl)benzenesulfonamide (0.5 g, 1.91 mmol) in dry CH3CN (20 ml), K2CO3 (0.8 g, 5.79 mmol), 2-bromo-1-(thiophen-2-yl) ethanone (0.5 g, 2.43 mmol) were added. The reaction mixture was stirred at room temperature for 6 h under N2 atmosphere. The solvent was removed and the residue was quenched with ice-water (50 ml), extracted with chloroform (3 *x* 10 ml) and dried (Na2SO4). Removal of solvent followed by the residue was dissolved in CH3CN (20 ml), Conc.HCl (3 ml) was added. The reaction mixture was then refluxed for 2 h. It was then poured over ice-water (50 ml), extracted with CHCl3 (3 *x* 10 ml) and dried (Na2SO4). Removal of solvent followed by crystallization from methanol afforded the compound as a colorless crystal.

### Refinement

H atoms were positioned geometrically and refined using riding model with C—H = 0.93Å and *U*_iso_(H) = 1.2Ueq(C).

### Crystal data

**Table:**

C_19_H_13_NO_3_S_2_	*Z* = 4
*M**_r_* = 367.42	*F*(000) = 760
Triclinic, *P*1	*D*_x_ = 1.466 Mg m^−^^3^
Hall symbol: -P 1	Mo *K*α radiation, λ = 0.71073 Å
*a* = 9.3605 (5) Å	Cell parameters from 6464 reflections
*b* = 10.8455 (5) Å	θ = 2.4–27.8°
*c* = 17.5482 (9) Å	µ = 0.34 mm^−^^1^
α = 88.716 (3)°	*T* = 295 K
β = 80.425 (2)°	Block, colourless
γ = 71.467 (2)°	0.35 × 0.25 × 0.20 mm
*V* = 1664.68 (15) Å^3^	

### Data collection

**Table:**

Bruker Kappa APEXII CCD diffractometer	8039 independent reflections
Radiation source: fine-focus sealed tube	6195 reflections with *I* > 2σ(*I*)
graphite	*R*_int_ = 0.040
Detector resolution: 0 pixels mm^-1^	θ_max_ = 28.0°, θ_min_ = 1.2°
ω and φ scans	*h* = −12→12
Absorption correction: multi-scan (*SADABS*; Sheldrick, 1996)	*k* = −14→14
*T*_min_ = 0.924, *T*_max_ = 0.951	*l* = −23→23
36289 measured reflections	

### Refinement

**Table:**

Refinement on *F*^2^	Primary atom site location: structure-invariant direct methods
Least-squares matrix: full	Secondary atom site location: difference Fourier map
*R*[*F*^2^ > 2σ(*F*^2^)] = 0.077	Hydrogen site location: inferred from neighbouring sites
*wR*(*F*^2^) = 0.245	H-atom parameters constrained
*S* = 1.07	*w* = 1/[σ^2^(*F*_o_^2^) + (0.092*P*)^2^ + 4.8598*P*] where *P* = (*F*_o_^2^ + 2*F*_c_^2^)/3
8039 reflections	(Δ/σ)_max_ < 0.001
453 parameters	Δρ_max_ = 0.55 e Å^−^^3^
0 restraints	Δρ_min_ = −0.37 e Å^−^^3^

### Special details

**Table:**

Geometry. All e.s.d.'s (except the e.s.d. in the dihedral angle between two l.s. planes) are estimated using the full covariance matrix. The cell e.s.d.'s are taken into account individually in the estimation of e.s.d.'s in distances, angles and torsion angles; correlations between e.s.d.'s in cell parameters are only used when they are defined by crystal symmetry. An approximate (isotropic) treatment of cell e.s.d.'s is used for estimating e.s.d.'s involving l.s. planes.
Refinement. Refinement of *F*^2^ against ALL reflections. The weighted *R*-factor *wR* and goodness of fit *S* are based on *F*^2^, conventional *R*-factors *R* are based on *F*, with *F* set to zero for negative *F*^2^. The threshold expression of *F*^2^ > σ(*F*^2^) is used only for calculating *R*-factors(gt) *etc*. and is not relevant to the choice of reflections for refinement. *R*-factors based on *F*^2^ are statistically about twice as large as those based on *F*, and *R*- factors based on ALL data will be even larger.

### Fractional atomic coordinates and isotropic or equivalent isotropic displacement parameters (Å^2^)

**Table:**

	*x*	*y*	*z*	*U*_iso_*/*U*_eq_	
C1	0.4481 (5)	0.1880 (4)	0.3523 (2)	0.0356 (8)	
C2	0.3808 (6)	0.3165 (4)	0.3334 (3)	0.0465 (10)	
H2	0.4309	0.3781	0.3332	0.056*	
C3	0.2370 (6)	0.3476 (5)	0.3150 (3)	0.0538 (12)	
H3	0.1889	0.4326	0.3021	0.065*	
C4	0.1597 (6)	0.2562 (5)	0.3149 (3)	0.0565 (13)	
H4	0.0614	0.2816	0.3031	0.068*	
C5	0.2277 (5)	0.1307 (5)	0.3320 (3)	0.0504 (11)	
H5	0.1768	0.0698	0.3314	0.061*	
C6	0.3763 (5)	0.0933 (4)	0.3507 (3)	0.0404 (9)	
C7	0.4734 (5)	−0.0266 (4)	0.3714 (3)	0.0431 (10)	
H7	0.4527	−0.1051	0.3736	0.052*	
C8	0.6014 (5)	−0.0084 (4)	0.3877 (3)	0.0376 (9)	
C9	0.7171 (5)	−0.0981 (4)	0.4277 (3)	0.0380 (9)	
C10	0.7561 (5)	−0.2365 (4)	0.4090 (2)	0.0366 (8)	
C11	0.7462 (5)	−0.2983 (4)	0.3425 (3)	0.0408 (9)	
H11	0.7074	−0.2553	0.3003	0.049*	
C12	0.8028 (6)	−0.4352 (5)	0.3473 (3)	0.0530 (12)	
H12	0.8071	−0.4930	0.3080	0.064*	
C13	0.8501 (6)	−0.4731 (5)	0.4156 (3)	0.0561 (13)	
H13	0.8880	−0.5597	0.4285	0.067*	
C14	0.7289 (5)	0.2305 (4)	0.4700 (3)	0.0383 (9)	
C15	0.8555 (5)	0.1793 (5)	0.5050 (3)	0.0493 (11)	
H15	0.9434	0.1191	0.4785	0.059*	
C16	0.8498 (6)	0.2187 (5)	0.5801 (3)	0.0552 (12)	
H16	0.9335	0.1830	0.6049	0.066*	
C17	0.7217 (6)	0.3102 (5)	0.6184 (3)	0.0537 (12)	
H17	0.7190	0.3373	0.6687	0.064*	
C18	0.5971 (6)	0.3618 (5)	0.5823 (3)	0.0493 (11)	
H18	0.5104	0.4238	0.6086	0.059*	
C19	0.5985 (5)	0.3231 (4)	0.5072 (3)	0.0442 (10)	
H19	0.5143	0.3583	0.4828	0.053*	
N1	0.5908 (4)	0.1243 (3)	0.3762 (2)	0.0371 (7)	
O2	0.8724 (4)	0.0828 (4)	0.3479 (2)	0.0562 (9)	
O1	0.6943 (5)	0.3007 (4)	0.3310 (2)	0.0583 (9)	
O3	0.7730 (4)	−0.0590 (3)	0.4761 (2)	0.0539 (9)	
S1	0.73502 (13)	0.18488 (11)	0.37382 (7)	0.0413 (3)	
S2	0.83327 (16)	−0.34647 (13)	0.47492 (8)	0.0544 (3)	
C20	0.3630 (5)	0.6980 (4)	0.1576 (2)	0.0421 (9)	
C21	0.2888 (7)	0.8279 (5)	0.1809 (3)	0.0569 (13)	
H21	0.3425	0.8867	0.1815	0.068*	
C22	0.1326 (7)	0.8653 (5)	0.2032 (3)	0.0645 (15)	
H22	0.0801	0.9519	0.2180	0.077*	
C23	0.0509 (6)	0.7792 (6)	0.2043 (3)	0.0642 (15)	
H23	−0.0542	0.8078	0.2212	0.077*	
C24	0.1237 (6)	0.6514 (6)	0.1808 (3)	0.0575 (13)	
H24	0.0684	0.5938	0.1807	0.069*	
C25	0.2827 (5)	0.6092 (5)	0.1569 (3)	0.0448 (10)	
C26	0.3888 (5)	0.4867 (4)	0.1316 (3)	0.0440 (10)	
H26	0.3660	0.4097	0.1281	0.053*	
C27	0.5280 (5)	0.5000 (4)	0.1135 (3)	0.0408 (9)	
C28	0.6673 (5)	0.4060 (4)	0.0691 (3)	0.0418 (9)	
C29	0.6939 (5)	0.2679 (4)	0.0865 (3)	0.0408 (9)	
C30	0.6482 (6)	0.2140 (5)	0.1540 (3)	0.0525 (12)	
H30	0.5864	0.2620	0.1974	0.063*	
C31	0.7070 (8)	0.0763 (6)	0.1495 (4)	0.0739 (17)	
H31	0.6879	0.0228	0.1894	0.089*	
C32	0.7944 (7)	0.0321 (5)	0.0798 (5)	0.0729 (18)	
H32	0.8415	−0.0558	0.0666	0.087*	
C33	0.7163 (5)	0.7268 (4)	0.0347 (3)	0.0405 (9)	
C34	0.8649 (6)	0.6794 (5)	−0.0023 (3)	0.0543 (12)	
H34	0.9382	0.6208	0.0221	0.065*	
C35	0.9052 (7)	0.7184 (6)	−0.0750 (4)	0.0673 (15)	
H35	1.0055	0.6852	−0.1006	0.081*	
C36	0.7982 (8)	0.8063 (6)	−0.1101 (3)	0.0643 (15)	
H36	0.8264	0.8334	−0.1594	0.077*	
C37	0.6495 (7)	0.8549 (5)	−0.0735 (3)	0.0593 (13)	
H37	0.5772	0.9144	−0.0979	0.071*	
C38	0.6077 (6)	0.8155 (5)	−0.0007 (3)	0.0500 (11)	
H38	0.5071	0.8483	0.0245	0.060*	
N2	0.5175 (4)	0.6304 (3)	0.1284 (2)	0.0415 (8)	
O4	0.6061 (5)	0.8001 (4)	0.1772 (2)	0.0609 (10)	
O5	0.7862 (4)	0.5782 (4)	0.1510 (2)	0.0586 (9)	
O6	0.7506 (4)	0.4410 (3)	0.0187 (2)	0.0563 (9)	
S3	0.66525 (14)	0.68399 (12)	0.13007 (7)	0.0450 (3)	
S4	0.80927 (17)	0.15141 (14)	0.01948 (9)	0.0630 (4)	

### Atomic displacement parameters (Å^2^)

**Table:**

	*U*^11^	*U*^22^	*U*^33^	*U*^12^	*U*^13^	*U*^23^
C1	0.038 (2)	0.033 (2)	0.034 (2)	−0.0084 (16)	−0.0079 (16)	0.0043 (15)
C2	0.052 (3)	0.034 (2)	0.048 (3)	−0.0044 (18)	−0.009 (2)	0.0024 (18)
C3	0.054 (3)	0.043 (3)	0.051 (3)	0.004 (2)	−0.012 (2)	0.006 (2)
C4	0.043 (3)	0.065 (3)	0.053 (3)	−0.001 (2)	−0.016 (2)	−0.005 (2)
C5	0.037 (2)	0.055 (3)	0.060 (3)	−0.012 (2)	−0.015 (2)	0.000 (2)
C6	0.038 (2)	0.038 (2)	0.046 (2)	−0.0112 (17)	−0.0076 (17)	−0.0050 (18)
C7	0.043 (2)	0.034 (2)	0.056 (3)	−0.0142 (18)	−0.0142 (19)	0.0015 (19)
C8	0.037 (2)	0.0271 (19)	0.048 (2)	−0.0079 (15)	−0.0099 (17)	0.0018 (16)
C9	0.035 (2)	0.036 (2)	0.042 (2)	−0.0094 (16)	−0.0083 (16)	0.0012 (17)
C10	0.038 (2)	0.0288 (19)	0.043 (2)	−0.0084 (15)	−0.0103 (17)	0.0036 (16)
C11	0.043 (2)	0.030 (2)	0.048 (2)	−0.0081 (17)	−0.0082 (18)	0.0000 (17)
C12	0.053 (3)	0.035 (2)	0.067 (3)	−0.007 (2)	−0.012 (2)	−0.006 (2)
C13	0.051 (3)	0.034 (2)	0.080 (4)	−0.009 (2)	−0.012 (2)	0.013 (2)
C14	0.040 (2)	0.032 (2)	0.046 (2)	−0.0149 (17)	−0.0091 (17)	−0.0033 (17)
C15	0.037 (2)	0.055 (3)	0.057 (3)	−0.0115 (19)	−0.012 (2)	−0.005 (2)
C16	0.053 (3)	0.057 (3)	0.062 (3)	−0.018 (2)	−0.025 (2)	−0.002 (2)
C17	0.067 (3)	0.053 (3)	0.050 (3)	−0.029 (2)	−0.014 (2)	−0.002 (2)
C18	0.048 (3)	0.042 (2)	0.055 (3)	−0.012 (2)	−0.004 (2)	−0.012 (2)
C19	0.038 (2)	0.037 (2)	0.056 (3)	−0.0097 (17)	−0.0105 (19)	−0.0022 (19)
N1	0.0380 (17)	0.0296 (16)	0.046 (2)	−0.0119 (14)	−0.0123 (15)	0.0019 (14)
O2	0.0428 (18)	0.064 (2)	0.058 (2)	−0.0176 (16)	0.0042 (15)	−0.0177 (17)
O1	0.075 (2)	0.056 (2)	0.059 (2)	−0.0395 (19)	−0.0159 (18)	0.0106 (17)
O3	0.060 (2)	0.0457 (18)	0.059 (2)	−0.0120 (16)	−0.0263 (17)	−0.0055 (15)
S1	0.0414 (6)	0.0411 (6)	0.0455 (6)	−0.0193 (4)	−0.0053 (4)	−0.0028 (4)
S2	0.0567 (7)	0.0483 (7)	0.0547 (7)	−0.0088 (5)	−0.0171 (6)	0.0126 (5)
C20	0.048 (2)	0.040 (2)	0.033 (2)	−0.0095 (18)	0.0001 (17)	0.0041 (17)
C21	0.064 (3)	0.039 (2)	0.055 (3)	−0.009 (2)	0.008 (2)	−0.003 (2)
C22	0.062 (3)	0.045 (3)	0.064 (3)	0.005 (2)	0.010 (3)	0.000 (2)
C23	0.047 (3)	0.063 (3)	0.063 (3)	0.000 (2)	0.009 (2)	0.010 (3)
C24	0.041 (3)	0.064 (3)	0.064 (3)	−0.016 (2)	−0.002 (2)	0.010 (3)
C25	0.042 (2)	0.045 (2)	0.047 (2)	−0.0137 (19)	−0.0083 (19)	0.0121 (19)
C26	0.048 (2)	0.037 (2)	0.047 (2)	−0.0145 (19)	−0.0047 (19)	0.0015 (18)
C27	0.043 (2)	0.036 (2)	0.042 (2)	−0.0111 (17)	−0.0076 (18)	0.0010 (17)
C28	0.040 (2)	0.043 (2)	0.041 (2)	−0.0105 (18)	−0.0063 (17)	−0.0031 (18)
C29	0.040 (2)	0.033 (2)	0.045 (2)	−0.0063 (17)	−0.0058 (17)	−0.0075 (17)
C30	0.061 (3)	0.040 (2)	0.049 (3)	−0.006 (2)	−0.006 (2)	0.001 (2)
C31	0.073 (4)	0.044 (3)	0.097 (5)	−0.010 (3)	−0.012 (3)	0.012 (3)
C32	0.058 (3)	0.039 (3)	0.115 (5)	−0.006 (2)	−0.013 (3)	−0.015 (3)
C33	0.043 (2)	0.040 (2)	0.043 (2)	−0.0207 (18)	−0.0083 (18)	0.0027 (18)
C34	0.043 (2)	0.059 (3)	0.059 (3)	−0.015 (2)	−0.005 (2)	0.001 (2)
C35	0.059 (3)	0.074 (4)	0.067 (4)	−0.027 (3)	0.009 (3)	−0.001 (3)
C36	0.085 (4)	0.066 (4)	0.048 (3)	−0.037 (3)	−0.002 (3)	0.006 (3)
C37	0.071 (4)	0.053 (3)	0.060 (3)	−0.021 (3)	−0.027 (3)	0.016 (2)
C38	0.046 (2)	0.052 (3)	0.054 (3)	−0.016 (2)	−0.011 (2)	0.005 (2)
N2	0.0423 (19)	0.0351 (18)	0.044 (2)	−0.0114 (15)	0.0004 (15)	−0.0031 (15)
O4	0.076 (3)	0.058 (2)	0.055 (2)	−0.0302 (19)	−0.0089 (18)	−0.0104 (17)
O5	0.055 (2)	0.064 (2)	0.061 (2)	−0.0171 (17)	−0.0240 (17)	0.0103 (18)
O6	0.055 (2)	0.051 (2)	0.054 (2)	−0.0133 (16)	0.0062 (16)	0.0019 (16)
S3	0.0491 (6)	0.0461 (6)	0.0439 (6)	−0.0193 (5)	−0.0107 (5)	−0.0003 (5)
S4	0.0559 (8)	0.0545 (8)	0.0683 (9)	−0.0081 (6)	0.0023 (6)	−0.0222 (7)

### Geometric parameters (Å, °)

**Table:**

C1—C2	1.393 (6)	C20—C21	1.394 (6)
C1—C6	1.398 (6)	C20—C25	1.399 (7)
C1—N1	1.423 (5)	C20—N2	1.410 (6)
C2—C3	1.371 (7)	C21—C22	1.377 (8)
C2—H2	0.9300	C21—H21	0.9300
C3—C4	1.401 (8)	C22—C23	1.381 (9)
C3—H3	0.9300	C22—H22	0.9300
C4—C5	1.357 (7)	C23—C24	1.373 (8)
C4—H4	0.9300	C23—H23	0.9300
C5—C6	1.411 (6)	C24—C25	1.403 (7)
C5—H5	0.9300	C24—H24	0.9300
C6—C7	1.409 (6)	C25—C26	1.410 (6)
C7—C8	1.350 (6)	C26—C27	1.342 (6)
C7—H7	0.9300	C26—H26	0.9300
C8—N1	1.422 (5)	C27—N2	1.414 (6)
C8—C9	1.472 (6)	C27—C28	1.483 (6)
C9—O3	1.216 (5)	C28—O6	1.216 (6)
C9—C10	1.458 (6)	C28—C29	1.472 (6)
C10—C11	1.390 (6)	C29—C30	1.373 (7)
C10—S2	1.717 (4)	C29—S4	1.710 (4)
C11—C12	1.415 (6)	C30—C31	1.417 (7)
C11—H11	0.9300	C30—H30	0.9300
C12—C13	1.357 (8)	C31—C32	1.356 (10)
C12—H12	0.9300	C31—H31	0.9300
C13—S2	1.693 (6)	C32—S4	1.678 (7)
C13—H13	0.9300	C32—H32	0.9300
C14—C15	1.378 (6)	C33—C34	1.371 (7)
C14—C19	1.382 (6)	C33—C38	1.381 (7)
C14—S1	1.757 (4)	C33—S3	1.757 (5)
C15—C16	1.382 (7)	C34—C35	1.367 (8)
C15—H15	0.9300	C34—H34	0.9300
C16—C17	1.371 (8)	C35—C36	1.365 (9)
C16—H16	0.9300	C35—H35	0.9300
C17—C18	1.375 (7)	C36—C37	1.372 (9)
C17—H17	0.9300	C36—H36	0.9300
C18—C19	1.388 (7)	C37—C38	1.371 (8)
C18—H18	0.9300	C37—H37	0.9300
C19—H19	0.9300	C38—H38	0.9300
N1—S1	1.674 (3)	N2—S3	1.666 (4)
O2—S1	1.417 (4)	O4—S3	1.423 (4)
O1—S1	1.427 (4)	O5—S3	1.424 (4)
			
C2—C1—C6	122.4 (4)	C21—C20—C25	121.7 (5)
C2—C1—N1	131.5 (4)	C21—C20—N2	131.5 (5)
C6—C1—N1	106.1 (3)	C25—C20—N2	106.8 (4)
C3—C2—C1	116.7 (5)	C22—C21—C20	117.1 (5)
C3—C2—H2	121.7	C22—C21—H21	121.5
C1—C2—H2	121.7	C20—C21—H21	121.5
C2—C3—C4	122.5 (5)	C21—C22—C23	122.4 (5)
C2—C3—H3	118.7	C21—C22—H22	118.8
C4—C3—H3	118.7	C23—C22—H22	118.8
C5—C4—C3	120.2 (5)	C24—C23—C22	120.5 (5)
C5—C4—H4	119.9	C24—C23—H23	119.8
C3—C4—H4	119.9	C22—C23—H23	119.8
C4—C5—C6	119.5 (5)	C23—C24—C25	119.0 (5)
C4—C5—H5	120.2	C23—C24—H24	120.5
C6—C5—H5	120.2	C25—C24—H24	120.5
C1—C6—C7	109.0 (4)	C20—C25—C24	119.3 (5)
C1—C6—C5	118.6 (4)	C20—C25—C26	108.2 (4)
C7—C6—C5	132.3 (4)	C24—C25—C26	132.5 (5)
C8—C7—C6	108.6 (4)	C27—C26—C25	108.4 (4)
C8—C7—H7	125.7	C27—C26—H26	125.8
C6—C7—H7	125.7	C25—C26—H26	125.8
C7—C8—N1	108.6 (4)	C26—C27—N2	109.3 (4)
C7—C8—C9	126.8 (4)	C26—C27—C28	127.3 (4)
N1—C8—C9	123.1 (4)	N2—C27—C28	122.2 (4)
O3—C9—C10	121.8 (4)	O6—C28—C29	122.1 (4)
O3—C9—C8	121.5 (4)	O6—C28—C27	121.6 (4)
C10—C9—C8	116.7 (4)	C29—C28—C27	116.2 (4)
C11—C10—C9	129.9 (4)	C30—C29—C28	129.0 (4)
C11—C10—S2	111.6 (3)	C30—C29—S4	111.7 (3)
C9—C10—S2	118.4 (3)	C28—C29—S4	119.1 (3)
C10—C11—C12	111.2 (4)	C29—C30—C31	111.7 (5)
C10—C11—H11	124.4	C29—C30—H30	124.1
C12—C11—H11	124.4	C31—C30—H30	124.1
C13—C12—C11	112.6 (5)	C32—C31—C30	111.7 (6)
C13—C12—H12	123.7	C32—C31—H31	124.2
C11—C12—H12	123.7	C30—C31—H31	124.2
C12—C13—S2	113.1 (4)	C31—C32—S4	113.5 (4)
C12—C13—H13	123.5	C31—C32—H32	123.3
S2—C13—H13	123.5	S4—C32—H32	123.3
C15—C14—C19	121.7 (4)	C34—C33—C38	120.3 (5)
C15—C14—S1	119.8 (4)	C34—C33—S3	120.2 (4)
C19—C14—S1	118.4 (3)	C38—C33—S3	119.3 (4)
C14—C15—C16	119.0 (5)	C35—C34—C33	119.9 (5)
C14—C15—H15	120.5	C35—C34—H34	120.1
C16—C15—H15	120.5	C33—C34—H34	120.1
C17—C16—C15	120.5 (5)	C36—C35—C34	120.0 (5)
C17—C16—H16	119.8	C36—C35—H35	120.0
C15—C16—H16	119.8	C34—C35—H35	120.0
C16—C17—C18	119.9 (5)	C35—C36—C37	120.6 (5)
C16—C17—H17	120.1	C35—C36—H36	119.7
C18—C17—H17	120.1	C37—C36—H36	119.7
C17—C18—C19	121.1 (5)	C38—C37—C36	119.9 (5)
C17—C18—H18	119.4	C38—C37—H37	120.1
C19—C18—H18	119.4	C36—C37—H37	120.1
C14—C19—C18	117.9 (4)	C37—C38—C33	119.4 (5)
C14—C19—H19	121.1	C37—C38—H38	120.3
C18—C19—H19	121.1	C33—C38—H38	120.3
C8—N1—C1	107.6 (3)	C20—N2—C27	107.2 (4)
C8—N1—S1	125.1 (3)	C20—N2—S3	126.1 (3)
C1—N1—S1	126.2 (3)	C27—N2—S3	125.3 (3)
O2—S1—O1	120.2 (2)	O4—S3—O5	119.6 (2)
O2—S1—N1	107.27 (19)	O4—S3—N2	105.7 (2)
O1—S1—N1	105.2 (2)	O5—S3—N2	107.4 (2)
O2—S1—C14	109.9 (2)	O4—S3—C33	108.0 (2)
O1—S1—C14	107.8 (2)	O5—S3—C33	109.9 (2)
N1—S1—C14	105.41 (19)	N2—S3—C33	105.2 (2)
C13—S2—C10	91.4 (2)	C32—S4—C29	91.5 (3)
			
C6—C1—C2—C3	1.9 (7)	C25—C20—C21—C22	−0.3 (8)
N1—C1—C2—C3	−177.6 (4)	N2—C20—C21—C22	176.6 (5)
C1—C2—C3—C4	0.0 (7)	C20—C21—C22—C23	1.4 (9)
C2—C3—C4—C5	−1.3 (8)	C21—C22—C23—C24	−1.9 (10)
C3—C4—C5—C6	0.7 (8)	C22—C23—C24—C25	1.2 (9)
C2—C1—C6—C7	179.1 (4)	C21—C20—C25—C24	−0.4 (7)
N1—C1—C6—C7	−1.2 (5)	N2—C20—C25—C24	−177.9 (4)
C2—C1—C6—C5	−2.5 (7)	C21—C20—C25—C26	−179.3 (5)
N1—C1—C6—C5	177.2 (4)	N2—C20—C25—C26	3.2 (5)
C4—C5—C6—C1	1.2 (7)	C23—C24—C25—C20	−0.1 (8)
C4—C5—C6—C7	179.1 (5)	C23—C24—C25—C26	178.5 (5)
C1—C6—C7—C8	1.4 (5)	C20—C25—C26—C27	−3.0 (5)
C5—C6—C7—C8	−176.6 (5)	C24—C25—C26—C27	178.3 (5)
C6—C7—C8—N1	−1.1 (5)	C25—C26—C27—N2	1.5 (5)
C6—C7—C8—C9	165.5 (4)	C25—C26—C27—C28	−165.7 (4)
C7—C8—C9—O3	−138.0 (5)	C26—C27—C28—O6	135.6 (5)
N1—C8—C9—O3	26.8 (7)	N2—C27—C28—O6	−30.2 (7)
C7—C8—C9—C10	39.3 (7)	C26—C27—C28—C29	−41.2 (7)
N1—C8—C9—C10	−155.9 (4)	N2—C27—C28—C29	153.0 (4)
O3—C9—C10—C11	−157.1 (5)	O6—C28—C29—C30	155.8 (5)
C8—C9—C10—C11	25.6 (7)	C27—C28—C29—C30	−27.5 (7)
O3—C9—C10—S2	20.2 (6)	O6—C28—C29—S4	−18.3 (6)
C8—C9—C10—S2	−157.1 (3)	C27—C28—C29—S4	158.4 (3)
C9—C10—C11—C12	177.3 (4)	C28—C29—C30—C31	−175.4 (5)
S2—C10—C11—C12	−0.1 (5)	S4—C29—C30—C31	−0.9 (6)
C10—C11—C12—C13	1.1 (6)	C29—C30—C31—C32	0.4 (8)
C11—C12—C13—S2	−1.7 (6)	C30—C31—C32—S4	0.4 (8)
C19—C14—C15—C16	2.0 (7)	C38—C33—C34—C35	−1.1 (8)
S1—C14—C15—C16	178.4 (4)	S3—C33—C34—C35	−176.6 (4)
C14—C15—C16—C17	−1.8 (8)	C33—C34—C35—C36	1.2 (9)
C15—C16—C17—C18	0.9 (8)	C34—C35—C36—C37	−0.8 (9)
C16—C17—C18—C19	−0.1 (8)	C35—C36—C37—C38	0.3 (9)
C15—C14—C19—C18	−1.2 (7)	C36—C37—C38—C33	−0.2 (8)
S1—C14—C19—C18	−177.7 (4)	C34—C33—C38—C37	0.5 (7)
C17—C18—C19—C14	0.3 (7)	S3—C33—C38—C37	176.2 (4)
C7—C8—N1—C1	0.3 (5)	C21—C20—N2—C27	−179.5 (5)
C9—C8—N1—C1	−166.9 (4)	C25—C20—N2—C27	−2.3 (5)
C7—C8—N1—S1	−168.3 (3)	C21—C20—N2—S3	13.4 (7)
C9—C8—N1—S1	24.5 (6)	C25—C20—N2—S3	−169.5 (3)
C2—C1—N1—C8	−179.8 (5)	C26—C27—N2—C20	0.5 (5)
C6—C1—N1—C8	0.6 (5)	C28—C27—N2—C20	168.5 (4)
C2—C1—N1—S1	−11.4 (7)	C26—C27—N2—S3	167.8 (3)
C6—C1—N1—S1	169.0 (3)	C28—C27—N2—S3	−24.2 (6)
C8—N1—S1—O2	27.6 (4)	C20—N2—S3—O4	9.0 (4)
C1—N1—S1—O2	−138.9 (4)	C27—N2—S3—O4	−156.0 (4)
C8—N1—S1—O1	156.7 (4)	C20—N2—S3—O5	137.7 (4)
C1—N1—S1—O1	−9.8 (4)	C27—N2—S3—O5	−27.2 (4)
C8—N1—S1—C14	−89.5 (4)	C20—N2—S3—C33	−105.2 (4)
C1—N1—S1—C14	104.0 (4)	C27—N2—S3—C33	89.9 (4)
C15—C14—S1—O2	7.8 (4)	C34—C33—S3—O4	119.1 (4)
C19—C14—S1—O2	−175.7 (3)	C38—C33—S3—O4	−56.5 (4)
C15—C14—S1—O1	−125.0 (4)	C34—C33—S3—O5	−13.0 (5)
C19—C14—S1—O1	51.6 (4)	C38—C33—S3—O5	171.3 (4)
C15—C14—S1—N1	123.1 (4)	C34—C33—S3—N2	−128.4 (4)
C19—C14—S1—N1	−60.4 (4)	C38—C33—S3—N2	56.0 (4)
C12—C13—S2—C10	1.4 (4)	C31—C32—S4—C29	−0.8 (5)
C11—C10—S2—C13	−0.7 (4)	C30—C29—S4—C32	1.0 (4)
C9—C10—S2—C13	−178.5 (4)	C28—C29—S4—C32	176.1 (4)

### Hydrogen-bond geometry (Å, °)

**Table:**

Cg is the centroid of the C20–C25 ring.

**Table:**

*D*—H···*A*	*D*—H	H···*A*	*D*···*A*	*D*—H···*A*
C2—H2···O1	0.93	2.33	2.879 (6)	117
C15—H15···O2	0.93	2.56	2.932 (6)	104
C21—H21···O4	0.93	2.33	2.878 (7)	117
C34—H34···O5	0.93	2.58	2.950 (7)	104
C17—H17···Cg8^i^	0.93	2.88	3.693 (6)	147

Symmetry codes: (i) −*x*+1, −*y*+1, −*z*+1.
